# Evaluation of a Novel Classification of Heat-Related Illnesses: A Multicentre Observational Study (Heat Stroke STUDY 2012)

**DOI:** 10.3390/ijerph15091962

**Published:** 2018-09-08

**Authors:** Takahiro Yamamoto, Motoki Fujita, Yasutaka Oda, Masaki Todani, Toru Hifumi, Yutaka Kondo, Junya Shimazaki, Shinichiro Shiraishi, Kei Hayashida, Shoji Yokobori, Shuhei Takauji, Masahiro Wakasugi, Shunsuke Nakamura, Jun Kanda, Masaharu Yagi, Takashi Moriya, Takashi Kawahara, Michihiko Tonouchi, Hiroyuki Yokota, Yasufumi Miyake, Keiki Shimizu, Ryosuke Tsuruta

**Affiliations:** 1 Advanced Medical Emergency and Critical Care Center, Yamaguchi University Hospital, 1-1-1 Minami-Kogushi, Ube, Yamaguchi 755-8505, Japan; tak-y@yamaguchi-u.ac.jp (T.Y.); todani-ygc@umin.ac.jp (M.T.); tsurutar@yamaguchi-u.ac.jp (R.T.); 2 Department of Acute and General Medicine, Yamaguchi University Graduate School of Medicine, 1-1-1 Minami-Kogushi, Ube, Yamaguchi 755-8505, Japan; motoki-ygc@umin.ac.jp; 3 Emergency and Critical Care medicine, St. Luke’s International Hospital, 9-1 Akashi-cho, Chuo-ku, Tokyo 104-8560, Japan; hifumitoru@gmail.com; 4 Department of Emergency Medicine and Critical Care Medicine, Juntendo University Urayasu Hospital, 2-1-1 Tomioka, Urayasu, Chiba 279-0021, Japan; kondokondou2000@yahoo.co.jp; 5 Department of Traumatology and Acute Critical Medicine, Osaka University Graduate School of Medicine, 2-15 Yamadaoka, Suita, Osaka 565-0871, Japan; jshimazaki@nifty.com; 6 Department of Emergency and Critical Care Medicine, Aizu Chuo Hospital, 1-1 Tsuruga-machi, Aizuwakamatsu, Fukushima 965-8611, Japan; shinshi@nms.ac.jp; 7 Department of Emergency and Critical Care Medicine, School of Medicine, Keio University, 35 Shinanomachi, Shinjuku-ku, Tokyo 160-8582, Japan; keilinda0714@gmail.com; 8 Department of Emergency and Critical Care Medicine, Nippon Medical School, 1-1-5 Sendagi, Bunkyo-ku, Tokyo 113-8603, Japan; shoji@nms.ac.jp (S.Y.); yokota@nms.ac.jp (H.Y.); 9 Department of Emergency Medicine, Asahikawa Medical University, Midorigaoka-higashi 2-1-1-1, Asahikawa, Hokkaido 078-8510, Japan; s-takauji@asahikawa-med.ac.jp; 10 Emergency and Critical Care Center, Toyama University Hospital, 2630, Sugitani, Toyama City, Toyama 930-0152, Japan; mwaka@med.u-toyama.ac.jp; 11 Department of Emergency Medicine, Wakayama Rosai Hospital, 93-1 Kinomoto, Wakayama City, Wakayama 640-8505, Japan; sns-nakamura@wakayamah.johas.go.jp; 12 Department of Emergency Medicine, Teikyo University School of Medicine, 2-11-1 Kaga, Itabashi-ku, Tokyo 173-8606, Japan; jkanda-cib@umin.ac.jp (J.K.); ymiyake-nsu@umin.ac.jp (Y.M.); 13 Department of Emergency and Critical Care Medicine, Urasoe General Hospital, 4-16-1 Iso, Urasoe, Okinawa 901-2132, Japan; masaharuy0130@gmail.com; 14 Department of Emergency and Critical Care Medicine, Saitama Medical Center, Jichi Medical University, 1-847 Amanuma-cho, Omiya-ku, Saitama City, Saitama 330-8503, Japan; tmoriya@jichi.ac.jp; 15 Japan Sport Council, 2-8-35 Kita-Aoyama, Minato-ku, Tokyo 107-0061, Japan; kawahara2630@gmail.com; 16 Japan Meteorological Business Support Center, To-nen Bld, 3-17 Kanda-Nishikicho, Chiyoda-ku, Tokyo 101-0054, Japan; tono@jmbsc.or.jp; 17 Emergency and Critical Care Center, Tokyo Metropolitan Tama Medical Centre, 2-8-29 Musashidai, Fuchu-shi, Tokyo 183-8524, Japan; icu240024@yahoo.co.jp

**Keywords:** heat-related illness, international classification, heat cramp, syncope, heat exhaustion, heat stroke, novel classification

## Abstract

The Japanese Association for Acute Medicine Committee recently proposed a novel classification system for the severity of heat-related illnesses. The illnesses are simply classified into three stages based on symptoms and management or treatment. Stages I, II, and III broadly correspond to heat cramp and syncope, heat exhaustion, and heat stroke, respectively. Our objective was to examine whether this novel severity classification is useful in the diagnosis by healthcare professionals of patients with severe heat-related illness and organ failure. A nationwide surveillance study of heat-related illnesses was conducted between 1 June and 30 September 2012, at emergency departments in Japan. Among the 2130 patients who attended 102 emergency departments, the severity of their heat-related illness was recorded for 1799 patients, who were included in this study. In the patients with heat cramp and syncope or heat exhaustion (but not heat stroke), the blood test data (alanine aminotransferase, creatinine, blood urea nitrogen, and platelet counts) for those classified as Stage III were significantly higher than those of patients classified as Stage I or II. There were no deaths among the patients classified as Stage I. This novel classification may avoid underestimating the severity of heat-related illness.

## 1. Introduction

The international classification for heat-related illnesses, which considers heat cramp and syncope, heat exhaustion, and heat stroke, has been used globally to assess the severity of heat-related illnesses [1,2]. In the international classification, heat stroke is defined as severe heat illness characterized by a core temperature above 40 °C and central nervous system (CNS) abnormalities. Heat exhaustion is defined as a mild to moderate heat illness caused by water or salt depletion, in which the core temperature may be normal, below normal, or slightly elevated (>37 °C but <40 °C) [1]. Thus, core temperature is one index used to evaluate the severity of heat stroke in the international classification.

The core temperatures of patients with heat-related illnesses have probably already begun to decrease in response to various factors before they are transferred to hospital [3,4], which could lead to a misdiagnosis or mismanagement. Therefore, the Japanese Association for Acute Medicine Committee recently proposed a novel classification system for the severity of heat-related illnesses with no reference to core temperature, to avoid underestimating the illness (Supplementary Figure S1) [5].

Stage I is any minor heat-related illness, including heat cramp and syncope. The signs and symptoms include dizziness, faintness, slight yawning, heavy sweating, muscle pain, and stiff muscles (muscle cramps). No impaired consciousness is observed.

Stage II is any heat-related illness not covered by Stage I or Stage III. The signs and symptoms include headache, vomiting, fatigue, a sinking feeling, reduced concentration, and impaired judgment.

Stage III refers to severe conditions in a patient with hyperthermia who has been under heat stress. Stage III patients show signs of brain dysfunction, such as loss of consciousness, cerebellar signs, or convulsive seizures. They also display liver, kidney, or blood clotting system dysfunction on blood tests. 

In practice, Stage I is a clinical condition that can be managed on site; Stage II is a clinical condition requiring immediate examination at a medical institution; and Stage III is a clinical condition requiring admission to hospital after blood sampling and assessment by medical workers (and depending on the case, intensive care). It is only permissible to restrict care to first aid and monitoring the patient when the Stage I symptoms are gradually improving. The patient should be taken to hospital immediately if Stage II symptoms occur or if no improvement in Stage I is observed (as assessed by someone other than the patient). Stage III must be confirmed by ambulance workers or by examination after arrival at hospital. Blood tests generally include measurement of the markers of renal (creatinine and blood urea nitrogen [BUN]), liver (aspartic aminotransferase [AST] and alanine aminotransferase [ALT]), or blood clotting system (platelet counts, D-dimer, prothrombin time [PT]) dysfunction, which show change at the early phase of organ dysfunction.

Although the symptoms observed at each level of severity are commonly observed symptoms, they do not always occur at the appropriate level of severity, and the illness should not be classified at a different level of severity if a symptom does not occur. The clinical status (severity) of heat stroke changes by the minute, depending on the timing and type of measures taken, and the condition of the patient. The purpose of this severity classification is to recognize abnormalities early, to expedite treatment and avoid serious consequences.

The novel classification broadly corresponds to the international classification (Stage I, heat cramp and syncope; Stage II, heat exhaustion; Stage III, heat stroke). However, the usefulness of the novel classification in assessing the severity of heat-related illness has not been fully validated. The possibility that Stage III patients with organ failure, according to the novel classification, may be included among patients diagnosed as having mild to moderate illness with the international classification system (heat cramp and syncope, and heat exhaustion) should be considered. In other words, it may be possible to underestimate severe heat-related illness in patients with organ failure when the international classification is used.

Our objective was to examine whether this novel classification system for the severity of heat-related illness has similar validity to the internationally accepted classification of heat-related illness and is useful in the diagnosis of patients with severe heat-related illness that includes organ failure.

## 2. Materials and Methods 

### 2.1. Study Overview

A prospective, multicentre, nationwide surveillance, observational study of heat-related illness (Heat Stroke STUDY 2012) was conducted by the Japanese Association for Acute Medicine Committee between 1 July and 30 September 2012, at 102 emergency hospitals in Japan (Appendix A). The study was approved by each hospital’s Institutional Review Board and was conducted in accordance with the ethical standards established in the 1964 Declaration of Helsinki and its later amendments (No. H25-31). The study data were collected from 1 July to 30 September 2012, the period with the highest average temperatures of the year in Japan. We used meteorological data measured by the Japan Meteorological Agency for Tokyo to clarify the heat trends in the summer of 2012 [6].

### 2.2. Data Collection

The study datasets were manually recorded by a staff member or medical doctor at each participating hospital using data entry sheets. We asked for information on patients who had been treated for a heat-related illness and the data entry sheets were submitted by mail. The data entry sheet was a simple form completed by each hospital with reference to each patient’s medical records. The items in the data entry sheets were categorized according to the patient characteristics, medical findings when attending an emergency department (ED), hospitalization, treatment content, disease severity according to the international and novel classification systems for heat-related illnesses, and outcomes. From these data, we extracted each patient’s age, sex, symptoms, physiological parameters, and laboratory data (ALT, creatinine, BUN, and platelet counts) when attending the ED, illness severity according to both classification systems, hospitalization (general ward or ICU), and outcome (survival or death at discharge). The international classification system defines the categories of heat cramp and syncope, heat exhaustion, and heat stroke [1,2]. The novel classification system defines Stage I, Stage II, and Stage III (Supplementary Figure S1) [5]. Although we used laboratory data (ALT, creatinine, BUN, and platelet count) as markers of hepatic, renal, and coagulation system dysfunction in this study, a specific cutoff value was not used to judge whether to hospitalize or discharge a patient. That decision was made by the attending physician at each hospital according to their overall judgment, based on the clinical conditions and laboratory results.

### 2.3. Statistical Analyses

Categorical variables were reported counts and percentages, and statistical testing was performed using the chi-square test. Quantitative variables were reported as median and interquartile ranges, and statistical testing was performed using non-parametric methods: the Mann-Whitney U test and the Kruskal-Wallis H test. P values obtained from multiple tests were adjusted using the Bonferroni method. Spearman’s rank correlation coefficient was calculated to determine the correspondence between the novel severity classification and the international classification, and the correlations between the blood test data (ALT, creatinine, BUN, and platelet counts) and each severity classification. To clarify the degree to which the severity of the heat-related illness was underestimated, the blood test data (ALT, creatinine, BUN, and platelet counts) were compared between the study patients classified with mild or moderate heat-related illness (but not heat stroke) according to the international classification and those classified with Stage I or II illness (but not Stage III) according to the novel classification. The utility of the data from patients classified with heat stroke or Stage III in predicting mortality was compared, to calculate the sensitivity and specificity of these parameters in predicting death, the positive likelihood ratio, and the negative likelihood ratio. A *p* value < 0.05 was considered statistically significant. All statistical analyses were performed with the statistical software SPSS Statistics version 19 (IBM, Armonk, NY, USA). 

## 3. Results

### 3.1. Changes in Air Temperature During the Study Period

The mean maximum temperature in Tokyo during the study period was 31.0 °C, and ranged from 21.9 °C (on 21 July 2012) to 35.7 °C (on 17 August 2012). The mean temperature and the mean minimum temperature were 27.2 °C and 24.4 °C, respectively. The total numbers of hot days and extremely hot days, defined by the Japan Meteorological Agency as having maximum temperatures exceeding 30.0 °C and 35.0 °C, respectively, were 64 days and 6 days. The mean temperature, mean maximum temperature, and mean minimum temperature over these 3 months (1 July to 30 September 2012) in the past 30 years were 25.9 °C, 29.5 °C and 23.0 °C, respectively [6].

### 3.2. Demographic Data of Patients with Heat-Related Illness

**Figure 1:**
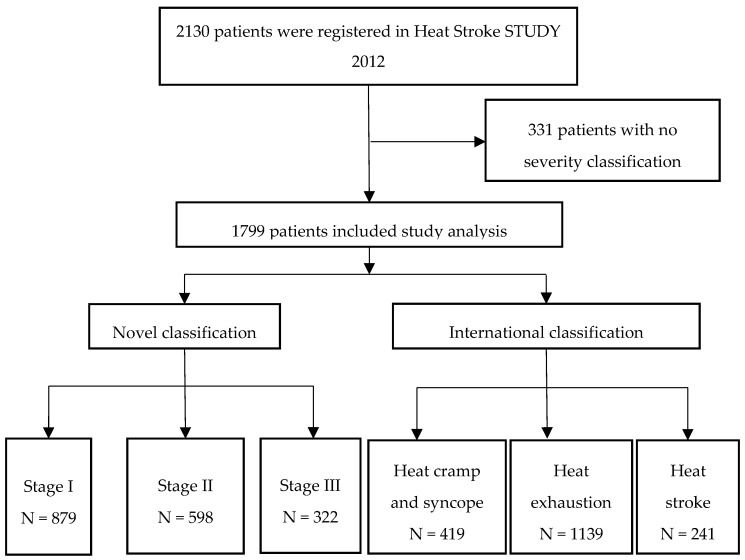
Flow chart of this study.

Between 1 July and 30 September 2012, 2130 patients were registered in the Heat Stroke STUDY 2012 at 102 EDs at the study hospitals (Appendix A). After the exclusion of 331 patients for whom the severity of their illness was not recorded with either the novel classification or the international classification, 1799 patients were included in the study. The numbers of heat cramp and syncope, heat exhaustion and heat stroke patients classified according to the international classification system, were 419, 1139 and 241, respectively, and the numbers of Stage I, II, and III patients, classified according to the novel classification system, were 879, 598 and 322, respectively (Figure 1). 

**Figure 2:**
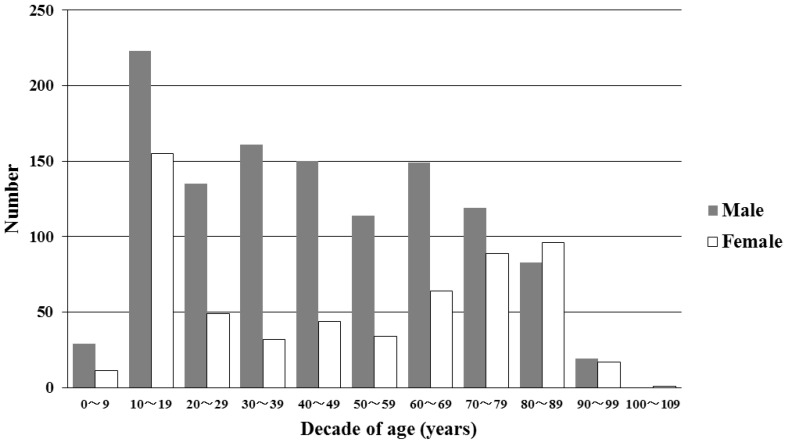
Numbers of patients with heat-related illness in 10-year age bins. Patient ages ranged from 1 to 102 years, and teenagers presented most frequently.

Table 1 lists the patient characteristics. The median patient age (interquartile ranges) was 44 (21–68) years. Thirty-seven deaths were recorded, 28 of which were caused by heat-related illness. More teenagers visited the EDs with heat-related illness than did any other age group (Figure 2).

### 3.3. Comparison of Heat-Related Illness Severity Diagnosed with the Novel Classification and the International Classification

**Figure 3:**
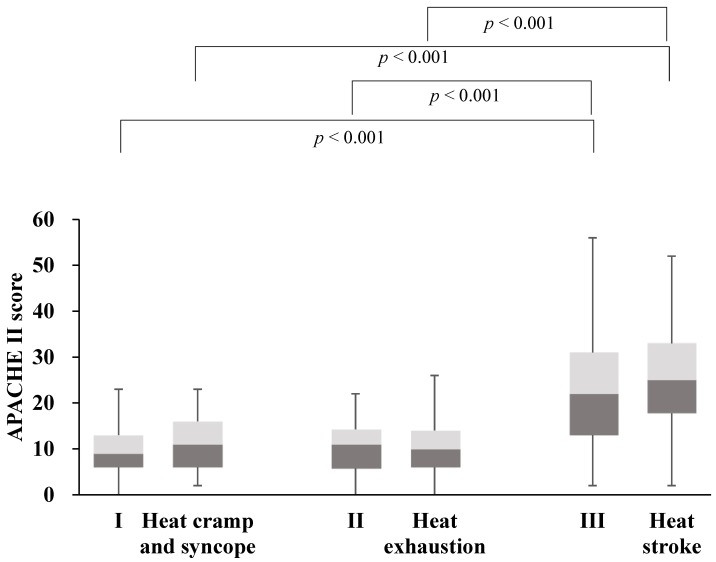
Acute Physiology and Chronic Health Evaluation (APACHE) II scores. Box-and-whisker plots comparing the APACHE II scores for patients classified with the international classification method or with our novel classification method. The box plots show the medians and interquartile ranges (difference between the first and third quartiles). The whiskers on the box plots indicate the maximum and minimum levels. Comparisons of the APACHE II scores among three degrees of severity in the novel classification and in the international classification were made with the Kruskal–Wallis *H* test. There was no significant difference between the two classification systems.

Figure 3 shows the acute physiology and chronic health evaluation (APACHE) II score for each classification. The score is obtained by adding the age and chronic disease score to the acute physiology score, which is the sum of the worst values recorded within the first 24 h of entering the ICU of a set of 12 physiological indicators. The APACHE II score correlates well with the outcome of critically ill patients [7]. The median APACHE II scores in Stages I, II, and III were 9 (6–13), 11 (6–14) and 22 (13–31), respectively, and in the heat cramp and syncope, heat exhaustion, and heat stroke groups were 11 (6–16), 10 (6–14) and 25 (18–33), respectively. Therefore, the APACHE II scores did not differ significantly between the novel classification and the international classification, although there was a significant difference between the scores for patients with Stage I or II illness and those for patients with Stage III illness in the novel classification, and between the scores for patients with heat cramp and syncope or heat exhaustion and those for patients with heat stroke in the international classification. 

**Figure 4:**
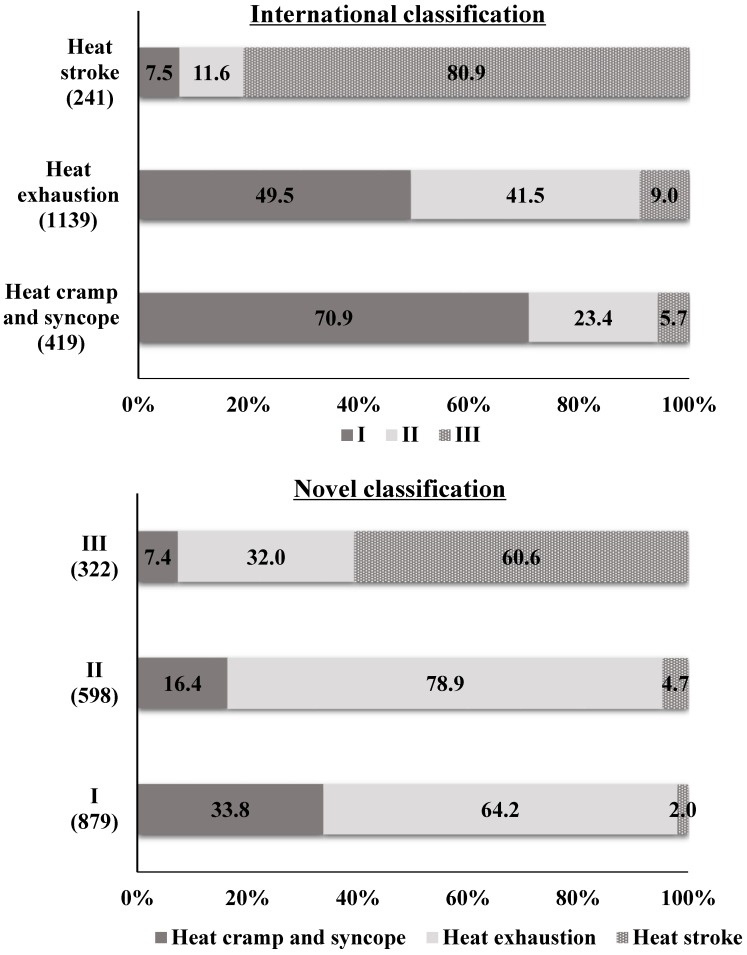
Distributions of the severity of heat-related illness according to the novel and international classifications. The distribution of severity determined with the novel classification correlated significantly with that determined with the international classification (Spearman’s rank correlation coefficient ρ = 0.448, *p* < 0.001, *n* = 1799).

Figure 4 shows the distributions of the severity of heat-related illness according to the novel classification and to the international classification. The distribution of severity determined with the novel classification correlated significantly with that determined with the international classification (ρ = 0.448, *p* < 0.001, *n* = 1799). However, of the 879 patients classified with Stage I illness, 564 (64.2%) had heat exhaustion, and 564 (49.5%) of the 1139 patients with heat exhaustion were classified as Stage I. The patients with heat cramp and syncope or heat exhaustion included some Stage III patients (heat cramp and syncope, 24 [5.7%]; heat exhaustion, 103 [9.0%]), and Stage I and II patients included a small number of patients with heat stroke (Stage I, 18 [2.0%]; Stage II, 28 [4.7%]).

### 3.4. Comparisons of Blood Test Data

Table 2 lists the Spearman’s rank correlation coefficients for the blood test data (ALT, creatinine, BUN, and platelet counts) and both severity classifications. In both classification systems, positive correlations were observed between the severity classification and ALT, creatinine, and BUN, and negative correlations between the severity classification and platelet count. The correlation coefficients for the novel classification were higher than those for the international classification.

Table 3 and Table 4 compare the blood test data (ALT, creatinine, BUN, and platelet counts) among patients with heat cramp and syncope or heat exhaustion (but not heat stroke) in the international classification (Table 3) and among patients with Stage I or II (but not Stage III) in the novel classification (Table 4). In the patients with heat cramp and syncope or heat exhaustion, the levels of ALT, creatinine, and BUN in the Stage III patients were significantly higher, and the platelet counts in Stage III patients were significantly lower than those in the Stage I and II patients. In contrast, in the Stage I and II patients, there were no significant differences in the parameters of the heat cramp and syncope or heat exhaustion patients and the heat stroke patients.

### 3.5. Outcomes of Patients with Heat-Related Illnesses

**Figure 5:**
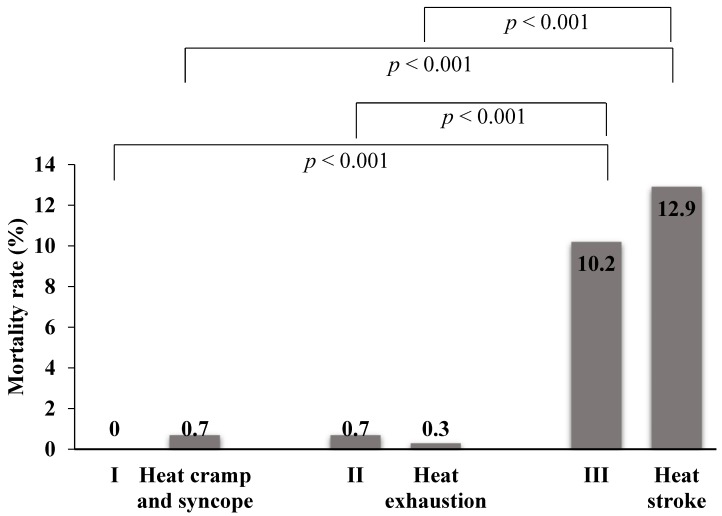
Mortality rate. There were no deaths among the patients classified in Stage I with the novel classification. The mortality rates for the three levels of severity according to the novel classification and the international classification were compared with a χ^2^ test. There was no significant difference between the two classifications.

Figure 5 shows the mortality rates according to the two classification systems. The mortality rates in Stages I, II and III were 0%, 0.7% and 10.2%, respectively, and in the patients with heat cramp and syncope, heat exhaustion, or heat stroke were 0.7%, 0.3% and 12.9%, respectively. The mortality rate was not significantly different between the novel classification and the international classification, but there was a significant difference between Stage I or II and Stage III in the novel classification, and between heat cramp and syncope or heat exhaustion and heat stroke in the international classification.

Table 5 shows the comparison of the data for patients with heat stroke and Stage III to predict mortality. Thirty–three (10.2%) of the patients with Stage III illness and 31 (12.9%) of those with heat stroke died. Therefore, the sensitivity and specificity of the classification systems in predicting death were 0.892 (95% CI: 0.755–0.957) and 0.836 (95% CI: 0.833–0.837), respectively, for patients with Stage III illness, and 0.838 (95% CI: 0.692–0.923) and 0.881 (95% CI: 0.878–0.883), respectively, for those with heat stroke. The positive and negative likelihood ratios (LR+) were 5.438 (95% CI: 4.526–5.884) and 0.129 (95% CI: 0.051–0.294), respectively, for the Stage III patients, and 7.030 (95% CI: 5.662–7.863) and 0.184 (95% CI: 0.087–0.351), respectively, for the heat stroke patients.

**Figure 6:**
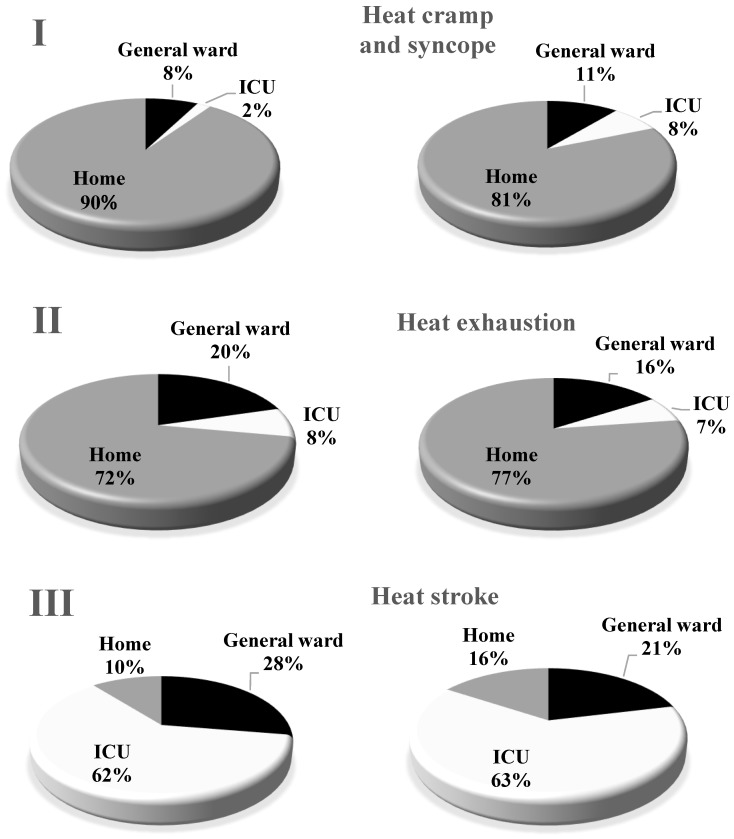
Management of patients with heat-related illness after leaving the emergency room. Home, general ward, and the ICU were assigned to 90%, 8% and 2% of Stage I patients, 81%, 11% and 8% of heat cramp and syncope patients, 72%, 20% and 8% of Stage II patients, 77%, 16% and 7% of heat exhaustion patients, 10%, 28% and 62% of Stage III patients, and 16%, 21% and 63% of heat stroke patients, respectively.

When the patients with heat illness left the emergency room (ER), 90%, 8% or 2% of the Stage I patients went home, to a general ward, or to the ICU, respectively. The corresponding figures were 81%, 11% or 8%, respectively, for the heat cramp and syncope patients; 72%, 20% or 8%, respectively, for the Stage II patients; 77%, 16% or 7%, respectively, for the heat exhaustion patients; 10%, 28% or 62%, respectively, for the Stage III patients, and 16%, 21% or 63%, respectively, for the heat stroke patients (Figure 6).

## 4. Discussion

In this study, the distribution of severity determined with the novel classification correlated significantly with that determined with the international classification. There was also no significant difference between the two classification systems in the distribution of APACHE II scores. These results demonstrate the similar validity of the novel and international classification systems in assessing heat-related illness. However, we found that Stage II and heat exhaustion, and Stage I and heat cramp and syncope did not correspond on a one-to-one basis. Moreover, patients with heat cramp and syncope or heat exhaustion included Stage III patients, and Stage I and II patients included patients with heat stroke. In a comparison of the blood test data, which included a hepatic enzyme, renal function biomarkers, and blood clotting system biomarker (ALT, creatinine, BUN, and platelet counts), Stage III patients who were classified with heat cramp and syncope or heat exhaustion (but not heat stroke) presented with significantly higher or lower levels. In contrast, there were no significant differences between heat cramp and syncope or heat exhaustion and heat stroke in Stage I and II patients (except for Stage III). These findings indicate that the illness severity in patients with organ failure may be underestimated by the international classification and overestimated by the novel classification. The sensitivity and specificity of the classification systems for predicting mortality were relatively high in patients classified with heat stroke in the international classification and as Stage III in the novel classification, so we consider that both these systems are useful in this regard.

The mean temperature, the mean maximum temperature, and the mean minimum temperature in Tokyo during the study period were higher than the average temperatures recorded in the last 30 years [6]. Therefore, the number of patients requiring emergency treatment for heat-related illness has increased in recent years [8].

In the novel classification system, three indicators of organ dysfunction are used to distinguish Stage II and III in the initial diagnosis: dysfunction of the CNS, liver or kidney dysfunction, and blood clotting system dysfunction (disseminated intravascular coagulation). The organs that can be functionally impaired in severe heat-related illness, such as the liver, kidney, and blood coagulation system, are taken to be the ‘target organs’. In the international classification, core temperature and brain dysfunction are used to distinguish heat stroke from heat exhaustion, with no reference to liver, kidney, or clotting system dysfunction [1].

The most severe heat-related illness is called ‘heat stroke’ in much of the literature. Heat stroke is defined clinically as a severe heat-related illness characterized by a core temperature exceeding 40 °C and CNS abnormalities, such as delirium, convulsions, or coma, resulting from exposure to environmental heat (classical heat stroke) or strenuous physical exercise (exertional heat stroke) [1]. According to this definition, consciousness disturbance and a core temperature over 40 °C are the main diagnostic criteria. However, to satisfy this definition, the heat-related illness must be extremely advanced. At the time of hospitalization for severe heat stroke, the core temperature of a patient with consciousness disturbance does not necessarily exceed 40 °C [3]. If too much emphasis is placed on a high core temperature (40 °C or more), an erroneous diagnosis may be made in the initial stage of the illness.

It has been noted for some time that core temperature measured at the site of collapse or in the emergency ward is inappropriate as a diagnostic criterion for heat-related illness [3]. Although the core temperature probably exceeds 42 °C at the moment of collapse, the patient’s temperature is rarely recorded on the spot before transportation to the ED. Furthermore, the first measurement recorded at the site of collapse may be made by untrained personnel and may be an axillary (cooled by wetting) or oral temperature (cooled by rapid respiration) rather than a rectal temperature [9]. The delay in temperature measurement and the inaccurate methods occasionally used mean that the first temperature recorded will be lower than that at the time of collapse [10]. Furthermore, the high core temperature immediately after the onset of heat stroke is alleviated to some extent by the cessation of exercise or labour and the transportation of the patient to a cool indoor setting during rescue [11]. Moreover, emergency medical staff begin the cooling process in the early phase of treatment when heat-related illness is suspected [4]. For these reasons, a temporary decline in core temperature to less than 40 °C may occur immediately after transportation to an emergency room (ER) or ED. Therefore, core temperature less than 40 °C at the time of transport to ER or ED does not necessarily exclude severe heat-related illness. Consequently, core temperature is not considered in the novel classification of heat-related illness.

Because organ failure can be a consequence of heat-related illness, it is especially important to detect such failure, including rhabdomyolysis, renal dysfunction, liver injury, and an increased systemic inflammatory response, in the very earliest phase of a heat-related illness [12,13]. Organ disorders that arise as a consequence of high temperature include muscle and gastrointestinal-tract dysfunction in mild cases. However, the CNS, circulation, liver, kidneys, and coagulation system can be impaired when a heat-related illness is severe [14]. Renal function is the most sensitive and practical biomarker in the early phase of heat-related illness, as it is easily affected by the burden of heat stress caused by dehydration and/or rhabdomyolysis [15,16]. Therefore, in the novel classification, in addition to brain dysfunction, the presence of renal, liver, or blood clotting system dysfunction is considered to be the most important criterion.

Although various blood test results, including creatine kinase [17,18,19] and procalcitonin levels [20,21,22], have been evaluated as indicators of the severity of heat-related illness, no adequate evidence of their utility has been obtained. An examination of 3227 cases of heat-related illness in Heat Stroke STUDY 2006, 2008, and 2010 in Japan [23,24,25] showed that scoring and summing the extent of damage to each organ (CNS, liver, kidney, and blood coagulation system), which defines Stage III in this novel classification of heat-related illnesses, may provide an effective index of severity and prognosis in cases of severe heat stroke. A novel early risk assessment tool for predicting clinical outcomes in patients with heat-related illnesses was recently reported based on data from the Heat Stroke STUDY [26]. Using this novel classification makes it possible to respond promptly and appropriately with the initial treatment for severe heat-related illness.

There were no deaths among patients classified with Stage I illness. This is an important point when considering the usefulness of the novel classification. More heat cramp and syncope patients were hospitalized after leaving the ER than Stage I patients, and more heat stroke patients than Stage III patients were sent home. This suggests that the international classification overestimates the severity of mild heat-related illness and underestimates the severity of serious heat-related illness.

A limitation of the novel classification system is that there is no clear standard by which to judge whether to hospitalize a patient with heat-related illness or send them home. In this study, the diagnosis of heat-related illness and the classification of its severity were made by the attending physician based on both the international and novel classifications.

Although prevention is the most important approach to heat stroke, the accurate early recognition of severe cases should allow early treatment and the prevention of death, even in patients with severe illness. This novel classification system for heat-related illnesses may improve the prognoses of patients with heat-related illness by simplifying the diagnostic criteria and recognizing heat-related illness as an important ‘syndrome’, thus avoiding the underestimation of severe cases.

## 5. Conclusions

This novel classification system for the severity of heat-related illnesses is consistent with the international classification. There were no deaths among patients classified with Stage I illness. Stage III patients included many with organ dysfunction who were classified as having mild to moderate illnesses, rather than heat stroke, in the international classification. Therefore, this novel classification system may circumvent the underestimation of the severity of heat-related illnesses.

## Figures and Tables

**Table 1:** Patient characteristics.

Variable	*n* = 1799
Age, years	44 (21–68)
Males	1184 (66)
Exertional heat illness	1121 (62)
APACHE II score	13 (8–22)
Laboratory data	
ALT (IU/L)	19.0 (14.0–32.0)
Creatinine (mg/dL)	0.90 (0.70–1.40)
BUN (mg/dL)	17.0 (12.7–23.0)
Platelet count (10^4^/μL)	23.0 (18.3–28.5)
Outcome	
Home	1259 (70)
General ward	278 (15)
ICU	262 (15)
Death	37 (2)

Data are expressed as the number (%) or median (interquartile range); APACHE: Acute Physiology and Chronic Health Evaluation; ALT: alanine aminotransferase; BUN: blood urea nitrogen; Reference values (as used at Yamaguchi University Hospital): creatinine, 0.65–1.07 mg/dL (male), 0.46–0.79 mg/dL (female); BUN, 8–20 mg/dL; ALT, 10–42 IU/L; platelet count, 15.8–34.8 × 10^4^/µL.

**Table 2:** Spearman’s rank correlation coefficients between blood test data (ALT, creatinine, BUN, and platelet count) and each of the severity classifications.

	International Classification	Novel Classification
ALT	0.147 *	0.214 *
Creatinine	0.183 *	0.306 *
BUN	0.147 *	0.256 *
Platelet count	−0.101 *	−0.165 *

ALT: alanine aminotransferase; BUN: blood urea nitrogen; * *p* value < 0.001.

**Table 3:** Comparison of blood test data among patients with heat cramp and syncope or heat exhaustion (but not heat stroke).

	Stage I and II	Stage III	*p* Value *
ALT (IU/L)	18.0 (13.0–29.0)	22.0 (15.0–43.0)	0.001
Creatinine (mg/dL)	0.83 (0.66–1.19)	1.25 (0.82–2.07)	<0.001
BUN (mg/dL)	16.0 (12.0–21.9)	20.0 (14.9–26.5)	<0.001
Platelet count (10^4^/μL)	23.6 (19.0–29.3)	21.5 (17.5–27.3)	0.003

Values are medians (interquartile ranges). * Mann–Whitney *U* test; ALT: alanine aminotransferase; BUN: blood urea nitrogen.

**Table 4:** Comparison of blood test data of patients classified as Stage I or II (but not Stage III).

	Heat Cramp and Syncope or Heat Exhaustion	Heat Stroke	*p* Value *
ALT (IU/L)	18.0 (13.0–29.0)	17.0 (13.0–24.0)	0.420
Creatinine (mg/dL)	0.83 (0.66–1.19)	0.88 (0.67–1.29)	0.487
BUN (mg/dL)	16.0 (12.0–21.9)	17.0 (14.3–22.9)	0.135
Platelet count (10^4^/μL)	23.6 (19.0–29.3)	23.2 (15.9–27.5)	0.122

Values are medians (interquartile ranges). * Mann–Whitney *U* test; ALT: alanine aminotransferase; BUN: blood urea nitrogen.

**Table 5:** Comparison of data between patients with heat stroke and Stage III in predicting mortality.

	III	Heat Stroke
Mortality, number (%)	33/322 (10.2)	31/241 (12.9)
Sensitivity for death (95% CI)	0.892 (0.755–0.957)	0.838 (0.692–0.923)
Specificity for death (95% CI)	0.836 (0.833–0.837)	0.881 (0.878–0.883)
LR+ (95% CI)	5.438 (4.526–5.884)	7.030 (5.662–7.863)
LR− (95% CI)	0.129 (0.051–0.294)	0.184 (0.087–0.351)

LR+: positive likelihood ratio; LR−: negative likelihood ratio; CI: confidence interval.

## Supplementary Materials

The following are available online at http://www.mdpi.com/1660-4601/15/9/1962/s1, Figure S1: Japanese Association of Acute Medicine Heat-Related Illness Classification 2015.

## Appendix A

**Facilities that participated in the Heat Stroke STUDY 2012**

1. Japanese Red Cross Kitami Hospital

2. Teine Keijinkai Hospital

3. Hachinohe City Hospital

4. Iwate Medical University Hospital

5. Ishinomaki Red Cross Hospital

6. Tohoku University Hospital

7. Akita University Hospital

8. Akita Red Cross Hospital

9. Yamagata Prefectural Central Hospital

10. Yamagata University Hospital

11. National Hospital Organization Mito Medical Center

12. Mito Saiseikai General Hospital

13. Dokkyo Medical University Hospital

14. Dokkyo Medical Universitsy Nikko Medical Center

15. Isesaki Municipal Hospital

16. Maebashi Red Cross Hospital

17. Saitama Medical University Hospital

18. Dokkyo Medical University Koshigaya Hospital

19. National Defence Medical College Hospital

20. Chiba University Hospital

21. Juntendo University Urayasu Hospital

22. Matsudo City Hospital

23. National Hospital Organization Disaster Medical Center

24. Nihon University Hospital

25. Tokai University Hachioji Hospital

26. Tokyo Medical University Hachioji Medical Center

27. Toho University Ohashi Medical Center

28. Tokyo Metropolitan Hiroo General Hospital

29. Nippon Medical School Tama Nagayama Hospital

30. Japanese Red Cross Medical Center

31. Kyorin University Hospital

32. Keio University Hospital

33. St. Luke's International Hospital

34. Teikyo University Hospital

35. The Jikei University Hospital

36. Toho University Omori Medical Center

37. Nippon Medical School Hospital

38. Nihon University Itabashi Hospital

39. St. Marianna University School of Medicine

40. Fujisawa City Hospital

41. National Hospital Organization Yokohama Medical Center

42. Yokohama City University Medical Center

43. Tokai University Hospital

44. Yamanashi Prefectural Central Hospital

45. University of Yamanashi Hospital

46. Nagano Red Cross Hospital

47. Saku Central Hospital Advanced Care Center

48. Aizawa Hospital

49. Kanazawa University Hospital

50. Ishikawa Prefectural Central Hospital

51. Gifu Prefectural General Medical Center

52. Gifu University Hospital

53. Takayama Red Cross Hospital

54. Gifu Prefectural Tajimi Hospital

55. Chuno Kosei Hospital

56. Shizuoka Saiseikai General Hospital

57. Numazu City Hospital

58. Seirei Mikatahara General Hospital

59. Seirei Hamamatsu General Hospital

60. Hamamatsu University School of Medicine, University Hospital

61. Ichinomiya Municipal Hospital

62. Daiyukai General Hospital

63. TOYOTA Memorial Hospital

64. Nagoya City University Hospital

65. Aichi Medical University Hospital

66. Okazaki City Hospital

67. Chukyo Hospital

68. Handa City Hospital

69. Ise Red Cross Hospital

70. Mie Prefectural General Medical Center

71. Saiseikai Shigaken Hospital

72. Nagahama Red Cross Hospital

73. Japanese Red Cross Society Kyoto Daini Hospital

74. Osaka University Hospital

75. Osaka Mishima Emergency Critical Care Center

76. National Hospital Organization Osaka National Hospital

77. Osaka Prefectural Nakakawachi Medical Center of Acute Medicine

78. Kobe University Hospital

79. Hyogo Emergency Medical Center

80. Hyogo Prefectural Kakogawa Medical Center

81. Kakogawa West City Hospital

82. Nara Medical University

83. Japanese Red Cross Wakayama Medical Center

84. Wakayama Medical University Hospital

85. Tottori University Hospital

86. Kawasaki Medical School Hospital

87. National Hospital Organization Kure Medical Center

88. Fukuyama City Hospital

89. Hiroshima Prefectural Hospital

90. National Hospital Organization Kanmon Medical Center

91. Tokuyama Central Hospital

92. Yamaguchi University Hospital

93. Tokushima Red Cross Hospital

94. Kagawa Prefectural Central Hospital

95. Kitakyushu City Yahata Hospital

96. St. Mary’s Hospital

97. Fukuoka University Hospital

98. Nagasaki University Hospital

99. Kumamoto Red Cross Hospital

100. National Hospital Organization Kumamoto Medical Center

101. Saiseikai Kumamoto Hospital

102. Naha City Hospital.
