# Supplementary material for: Evaluation of a Novel Classification of Heat-Related Illnesses: A Multicentre Observational Study (Heat Stroke STUDY 2012)
Source: Int J Environ Res Public Health. 2018 Sep 8;15(9):1962. doi: 10.3390/ijerph15091962 (PMC6165559; doi:10.3390/ijerph15091962)
Supplement: Supplementary file 1 [file ijerph-15-01962-s001.zip › ijerph-345442-supplementary/ijerph-345442-supplementary.pptx]

## Slide 1
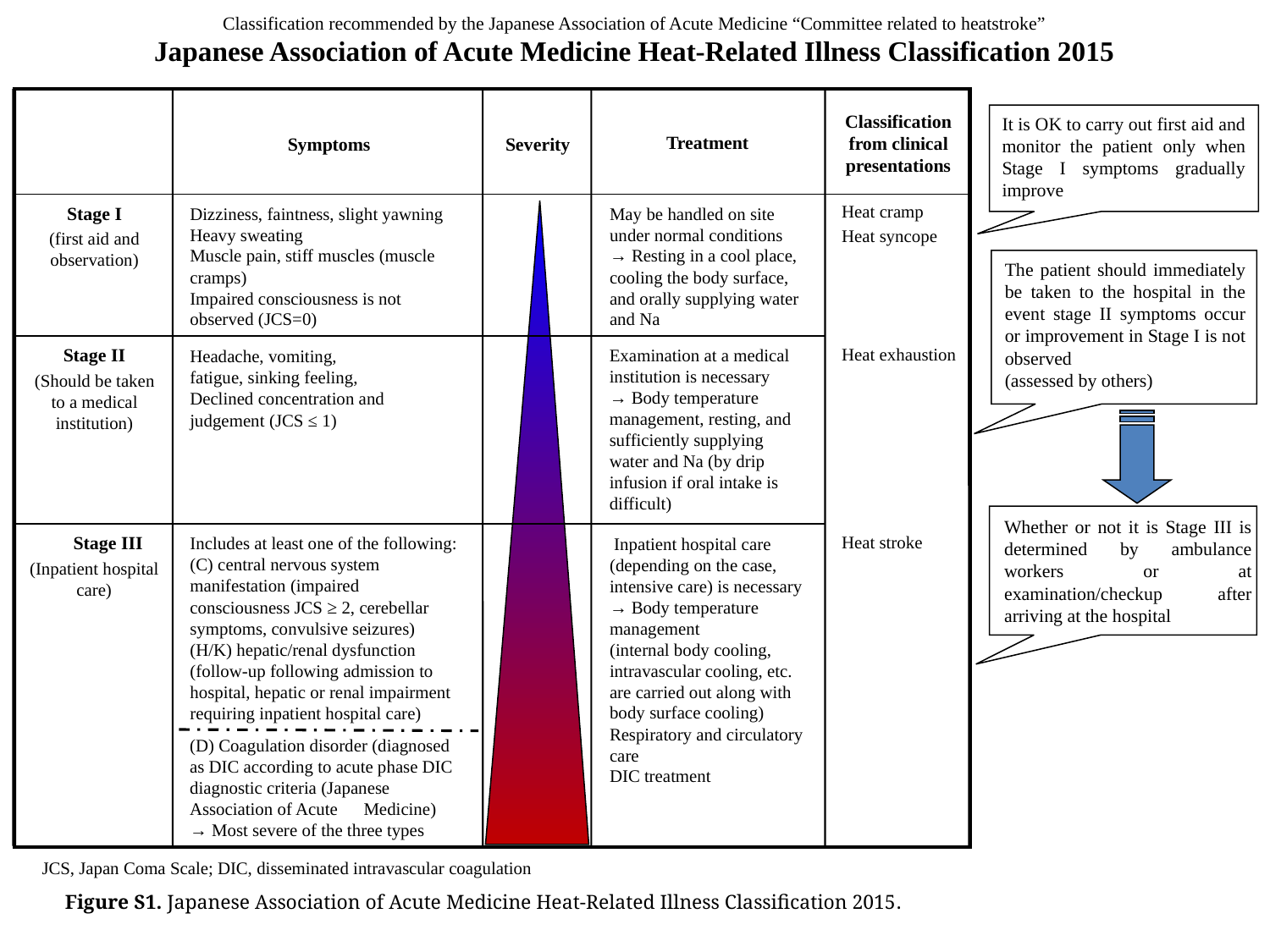

Classification recommended by the Japanese Association of Acute Medicine “Committee related to heatstroke”
Japanese Association of Acute Medicine Heat-Related Illness Classification 2015
Symptoms
Severity
Treatment
Classification from clinical presentations
It is OK to carry out first aid and monitor the patient only when Stage I symptoms gradually improve
Heat cramp
Heat syncope
Dizziness, faintness, slight yawning
Heavy sweating
Muscle pain, stiff muscles (muscle cramps)
Impaired consciousness is not observed (JCS=0)
May be handled on site under normal conditions
→ Resting in a cool place, cooling the body surface, and orally supplying water and Na
Stage I
(first aid and observation)
The patient should immediately be taken to the hospital in the event stage II symptoms occur or improvement in Stage I is not observed
(assessed by others)
Stage II
(Should be taken to a medical institution)
Heat exhaustion
Examination at a medical institution is necessary
→ Body temperature management, resting, and sufficiently supplying water and Na (by drip infusion if oral intake is difficult)
Headache, vomiting,
fatigue, sinking feeling,
Declined concentration and judgement (JCS ≤ 1)
Whether or not it is Stage III is determined by ambulance workers or at examination/checkup after arriving at the hospital
Heat stroke
Includes at least one of the following:
(C) central nervous system manifestation (impaired consciousness JCS ≥ 2, cerebellar symptoms, convulsive seizures)
(H/K) hepatic/renal dysfunction (follow-up following admission to hospital, hepatic or renal impairment requiring inpatient hospital care)
 Inpatient hospital care (depending on the case, intensive care) is necessary
→ Body temperature management
(internal body cooling, intravascular cooling, etc. are carried out along with body surface cooling)
Respiratory and circulatory care
DIC treatment
　 Stage III
(Inpatient hospital care)
(D) Coagulation disorder (diagnosed as DIC according to acute phase DIC diagnostic criteria (Japanese Association of Acute　Medicine)
→ Most severe of the three types
JCS, Japan Coma Scale; DIC, disseminated intravascular coagulation
Figure S1. Japanese Association of Acute Medicine Heat-Related Illness Classification 2015.
